# The mitogenomes of *Gelochelidon nilotica* and *Sterna hirundo* (Charadriiformes, Sternidae) and their phylogenetic implications

**DOI:** 10.1080/23802359.2017.1372709

**Published:** 2017-09-04

**Authors:** Chao Yang, Qing-Xiong Wang, Xue-Juan Li, Hao Yuan, Hong Xiao, Yuan Huang

**Affiliations:** a Shaanxi Institute of Zoology, Xi’an, China;; b School of Life Sciences, Shaanxi Normal University, Xi’an, China

**Keywords:** *Gelochelidon nilotica*, *Sterna hirundo*, mitogenome, structure, phylogeny

## Abstract

*Gelochelidon nilotica* and *Sterna hirundo* are two sympatric breeding species. The mitogenomes of *G. nilotica* and *S. hirundo* are 16,748 bp and 16,707 bp in size. Both two mitogenomes reveal the same gene order and genomic organization to that of typical avian mtDNA. The first conserved blocks with a interrupted poly-C are present in the two species control regions, but not existed in *S. albifrons*. Seventeen and 11 simple sequence repeats are found in *G. nilotica* and *S. hirundo*, respectively. Phylogenetic analysis indicated that Sternidae has the closest relationship with Laridae. We supported that Stercorariidea is a sister group to (Alcidae (Laridae, Sternidae)), *G. nilotica* is genetically most related to *S. hirundo* (all belonged to Black cap species), but distant with *S. albifrons* (White blaze species) in kinship, and suggested that the status of *Larus vegae* should be further investigated.

The terns (Charadriiformes: Sternidae) are a distinctive group of seabirds that occupy aquatic environments the world over and demonstrate an interesting array of variations on a life history centred around aquatic foraging and colonial nesting. Gull-billed Tern (*Gelochelidon nilotica*) and Common Tern (*Sterna hirundo*) belonging to Sternidae, are widely distributing coastal seabirds and can be found breeding in most of Europe, Asia and North America, and both of their Northern populations making long-distance southwards migratory movements. The two species both have extremely large range and population size, and are evaluated as Least Concern (IUCN 2016), but they are vulnerable to the deterioration and loss of habitat, human disturbance, pesticide pollution, agricultural intensification, fluctuating water levels (del Hoyo et al. 1996), beach erosion and the development or modification of foraging sites (Molina and Erwin 2006) during the breeding season, and they also may be threatened by future outbreaks of the virus (Melville and Shortridge 2006). The complete mitogenome is an important marker for studies related to taxonomy, biodiversity and conservation (Anmarkrud and Lifjeld 2017). Using complete mitogenomes, one can analyse nucleotide variation, obtain information on haplotypes, and elucidate current population structures of species (Yamamoto et al. 2005). Nevertheless, in terms of phylogenetics, the limited molecular data dampen the diversity and evolution studies in interspecific of Sternidae.

Naturally dead *G. nilotica* and *S. hirundo* chicks were collected during the breeding season at Hongjian Nur (39°04′N, 109°53′E), Shaanxi, China. The specimens (proof number: X08, A07) were deposited in the animal specimens museum of Shaanxi Institute of Zoology, Xi’an, China. The mitogenomes were determined after PCR amplification, sequencing and annotation based on previously published sequence (Yang et al. 2016a).

The total length of the *G. nilotica* (Genbank: MF582631) and *S. hirundo* (Genbank: MF582632) mitochondrial genomes is 16,748 bp and 16,707 bp, and with the base composition A + T are 56.0% and 55.8%, respectively. Their genes’ arrangement and orientation are identical to that of typical avian mtDNA (Gibb et al. 2007).

The 13 PCGs of *G. nilotica* and *S. hirundo* are similar to that observed for other Charadriiformes. The typical ATN (ATG or ATC) start codons are present in *G. nilotica* and *S. hirundo* PCGs, with the exception of the *COI* in two species, which utilize GTG as start codons. Open-reading frames of most PCGs end with AGG, AGA, TAA, or TAG, while *COIII* and *ND4* have the incomplete stop codon T in two species. The *ND3* genes of *G. nilotica* and *S. hirundo* are all with an extra C nucleotide in 174 sites.

The *srRNA* and *lrRNA* in *G. nilotica* and *S. hirundo* are 967, 1592 bp and 967, 1596 bp, respectively. All tRNA genes in two species, ranging from 66 to 74 bp in size, can fold into typical cloverleaf secondary structures, with exception of the shortest *tRNA^Ser^(AGN)* (66 bp), in which the DHU arm has been replaced by a simple loop. The control regions are 1198 bp and 1154 bp in length in *G. nilotica* and *S. hirundo*. Especially, the interrupted poly-C sequences (5′-**CCCCCCCCCTACCCCCCCACCCCCC**-3′, bp positions: 15,560–15,584; 5′-**CCCCCCCCCCTTCCCCCCCC**-3′, bp positions: 15,563–15,582) in domain I of control regions are present in *G. nilotica* and *S. hirundo*, but no relevant structure is found in *S. albifrons*. This sequence is similar to that in the ‘goose hairpin’ in Laridae (Yang et al. 2016b) and to variants found in *Himantopus himantopus* (Yang et al. 2017). Seventeen simple sequence repeats with 5′-**ACAACAA**-3′ (bp positions: 16,623–16,741) and 11 repeats with 5′-**ACAAACA**-3′ (bp positions: 16,624–16,700) are existed in *G. nilotica* and *S. hirundo*, respectively.

To investigate the phylogenetic positions of *G. nilotica* and *S. hirundo* (Sternidae), Bayesian inference (BI) tree and maximum likelihood (ML) tree were reconstructed based on 41 mitogenomes PCGs using MrBayes ver. 3.2.2 (Ronquist et al. 2012) and RAxML (Stamatakis 2006) under the best partitioned scheme and optimal model analysed in Partitionfinder v1.1.1 (Lanfear et al. 2012) (Models GTR + I+G and GTR + G). *Gallirallus philippensis* and *Grus vipio* were selected as outgroups. The phylograms obtained from BI and ML (data not shown) were all strongly indicated that Sternidae was a sister group to Laridae (Thomas et al. 2004; Livezey 2010). The first analysis was supported between (Stercorariidea (Alcidae (Laridae, Sternidae))) (bootstrap value 1.00 in BI) and ((Stercorariidea, Alcidae) (Laridae, Sternidae)) (bootstrap value 0.87 in ML) (Maxwell and Harrison 2008). It was also supported that *S. hirundo* should be belonged to Black cap species with *G. nilotica*, *S. albifrons* categorized into the White blaze species (Bridge et al. 2005). Extraordinarily, the *Larus vegae* was more primitive and located in the root of the tree, but not belonged to the branch of Laridae (Figure 1).

**Figure 1.**
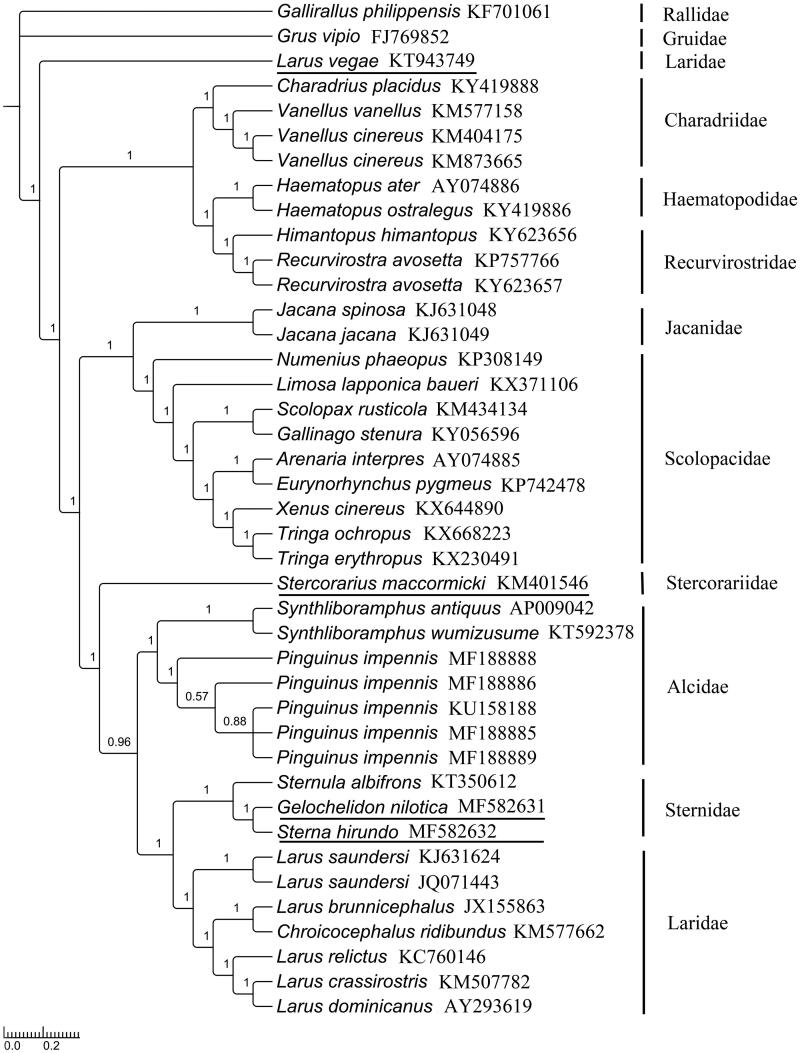
Topology of Bayesian tree for 41 species based on mitogenome PCG sequences. GenBank accession numbers are indicated following species name (numbers on nodes are bootstrap values).
